# Correction

**DOI:** 10.1080/07853890.2024.2346423

**Published:** 2024-05-03

**Article title:** A cross-sectional study of nutritional status in healthy, young, physically- active German omnivores, vegetarians and vegans reveals adequate vitamin B12 status in supplemented vegans

**Authors:** Maximilian Andreas Storz, Alexander Müller, Lisa Niederreiter, Amy M. Zimmermann-Klemd, Martin Suarez-Alvarez, Stefanie Kowarschik, Monique Strittmatter, Evelyn Schlachter, Cristian Pasluosta, Roman Huber and Luciana Hannibal

**Journal:** *Annals of Medicine*

**Bibliometrics:** Volume 55, Number 02, page 2269969

**DOI:** 10.1080/07853890.2023.2269969

When this article was first published online, the below listed tabulated data were incorrectly mentioned in the article.

- In **Table 2**, the *p*-value for the intergroup difference of vitamin E intake was listed as < 0.001. This has been corrected as *p* = 0.177. The error in *p*-value for vitamin E was not carried over into the main text and hence the interpretations on vitamin E remain correct as published.
- **Table 3**:
  - (a) The vitamin B12 supplement intake frequency in the vegan group was given as 89.47% (*n* = 34/38 participants), which has been corrected as 92.11% (*n* = 35/38 participants). Correspondingly, the number of vegans **not** supplementing vitamin B12 is *n* = 3 (7.89%). The overall *p*-value remains unaltered as *p* < 0.001.
  - (b) The general supplement intake frequency in the vegan group was reported as 86.84% (*n* = 33/38 participants), which has been corrected as 94.74% (*n* = 36/38 participants). Correspondingly, the number of vegans **not** taking supplements is *n* = 2 (5.26%). The overall *p*-value remains insignificant (0.090). These frequencies were mentioned in the text, as follows:
    - **Abstract:** “Fewer lacto-ovo-vegetarians used B12 supplements compared to vegans (51% versus 90%)”. This should have read: 51% versus 92%.
    - **Results**: Page 6, “Supplementation behavior”: “A total of 77.5–86.8% of participants took supplements in each group”. This should have read: 77.5–94.74%.
    - **Results**: Page 6, “Supplementation behavior”: “The number of participants supplementing vitamin B12 was highest in vegans (89.5%), as well.” This should have read: (92%). This frequency counting error affected only the above-mentioned proportions. Intake frequencies and dosages remain correct as published.
  - (c) We reported a median docosahexaenoic acid supplement intake in the lacto-ovo-vegetarian group of 185 (330) mg. However, the correct dosage is 441 (469) mg. The *p*-value remains unaffected.
- **Table 4**:
  - (a) The standard deviation for red blood cell count in the lacto-ovo-vegetarian group was listed as 4.5 ± 0.05. This has been corrected as 4.55 ± 0.48.
  - (b) The *p*-value for parathyroid hormone in serum was listed as *p* = 0.093. This has been corrected as *p* = 0.83. The interpretation remains correct as published.

**Table 2.** Nutrient intake data of study participants by dietary group.

	Omnivores (*n* = 40)	Lacto-Ovo-Vegetarians (*n* = 37)	Vegans (*n* = 38)	*p*-value
Energy intake (kcal/d)	1995.14 (938.71)	2085.86 (692.42)	2068.29 (881.86)	0.819^a^
Carbohydrate intake (g/d)	242.71 (101.94)	241.75 (122.73)	256.48 (140.863)	0.193^a^
Protein intake (g/d)	73.66 (45.43)	65.88 (22.80)	62.33 (39.64)	0.297^a^
Fat intake (g/d)	81.39 (49.26)	75.85 (35.77)	83.92 (31.58)	0.981^a^
Fiber intake (g/d)	24.21 (20.14)	31.2 (14.75)	33.61 (19.54)	**0.001**^a^
Alcohol intake (g/d)	0.062 (4.95)	0.08 (2.82)	0.028 (2.76)	0.891^a^
Cholesterol intake (mg/d)	177.93 (224.77)	70.85 (154.58)	17.75 (49.40)	**<0.001**^a^
Uric acid intake (mg/d)	257.65 (134.37)	234.56 (104.29)	259.72 (191.70)	0.451^a^
Polyunsaturated fatty acid intake (g/d)	10.20 (10.91)	10.42 (12.2)	13.10 (11.231)	0.194^a^
Monounsaturated fatty acid intake (g/d)	21.90 (17.66)	18.51 (14.81)	18.39 (13.74)	0.320^a^
Saturated fatty acid intake (g/d)	31.97 (18.71)	23.00 (14.33)	18.55 (9.61)	**<0.001**^a^
Sodium intake (mg/d)	2102.30 (1174.06)	1846.57 (722.31)	2257.65 (1199.241)	0.087^a^
Potassium intake (mg/d)	2079.19 (1642.59)	2090.95 (689.273)	2549.37 (1114.57)	0.077^a^
Calcium intake (mg/d)	541.24 (448.97)	571.70 (370.56)	475.38 (198.13)	0.259^a^
Phosphorus intake (mg/d)	788.27 (489.44)	798.65 (400.25)	664.14 (529.19)	0.288^a^
Magnesium intake (mg/d)	246.76 (177.96)	270.62 (180.05)	312.96 (169.47)	0.053^a^
Iron intake (mg/d)	7.83 (4.45)	9.22 (4.24)	10.39 (5.87)	0.142^a^
Vitamin A intake (mg/d)	0.29 (0.30)	0.23 (0.22)	0.07 (0.08)	**<0.001**^a^
Vitamin C intake (mg/d)	103.21 (67.19)	139.94 (93.96)	172.41 (132.71)	**<0.001**^a^
Vitamin D intake (μg/d)	2.07 (3)	1.24 (1.37)	0.92 (0.97)	**<0.001**^a^
Vitamin E intake (mg/d)	9.74 (9.80)	10.90 (11.64)	13.39 (10.26)	0.177^a^
Vitamin B1 intake (mg/d)	0.66 (0.56)	0.84 (0.63)	1.02 (1.05)	0.059^a^
Vitamin B2 intake (mg/d)	0.81 (0.54)	0.77 (0.38)	0.86 (0.38)	0.680^a^
Niacin intake (niacin equivalents, μg/d)	18,448.72 (12511.15)	14,047.95 (5471.74)	13993.22 (6421.36)	**0.047^a^**
Vitamin B6 intake (mg/d)	1.30 ± 0.58	1.24 ± 0.48	1.54 ± 0.61	**0.046**^b^
Vitamin B12 intake (μg/d)	2.14 (2.29)	0.98 (1.34)	0.43 (0.58)	**<0.001**^a^
Folic acid intake (μg/d)	224.39 (138.74)	241.92 (114.62)	287.76 (149.34)	**0.025**^a^
Zinc intake (mg/d)	6.41 (4.30)	6.37 (2.92)	5.33 (3.61)	0.727^a^
Iodine intake (μg/d)	54.03 (39.71)	38.14 (33.74)	37.88 (20.18)	**0.030**^a^

Normally distributed data is shown with its mean ± standard deviation; not normally distributed data is shown with its median and IQR in parenthesis.

^a^Based on Kruskal–Wallis *H* test.

^b^Based on analysis of variance. Nutrient intakes given in this table are from foodstuffs only and do not include nutrients taken in the form of supplements. Nutrient intakes from supplements are provided in Table 3.

**Table 3.** Supplementation characteristics of study participants by dietary group.

	Omnivores (*n* = 40)	Lacto-Ovo-Vegetarians (*n* = 37)	Vegans (*n* = 38)	*p*-value
Supplement intake				.090^a^
Yes	*n* = 31 (77.5%)	*n* = 30 (81.08%)	*n* = 36 (94.74%)	
No	*n* = 9 (22.5%)	*n* = 7 (18.92%)	*n* = 2 (5.26%)	
Multivitamin supplement intake				**.045**^a^
Yes	*n* = 9 (22.5%)	*n* = 10 (27.03%)	*n* = 18 (47.37%)	
No	*n* = 31 (77.5%)	*n* = 27 (72.97%)	*n* = 20 (52.63%)	
Vitamin B 12 supplement intake				**<.001**^a^
Yes	*n* = 11 (27.5%)	*n* = 19 (51.35%)	*n* = 35 (92.11%)	
No	*n* = 29 (72.5%)	*n* = 18 (48.65%)	*n* = 3 (7.89%)	
Dosage (μg)	25 (335.5)	400 (977.5)	250 (980)	.136^b^
Frequency (day/year)	180 (287)	40 (144)	365 (209)	**.001**^b^
Vitamin D supplement intake				.561^a^
Yes	*n* = 25 (62.5%)	*n* = 20 (54.05%)	*n* = 25 (65.79%)	
No	*n* = 15 (37.5%)	*n* = 17 (45.95%)	*n* = 13 (34.21%)	
Dosage (IU)	1000 (4600)	2350 (2200)	2000 (4000)	.753^b^
Frequency (day/year)	156 (313)	90 (335)	365 (235)	.083^b^
Iron supplement intake				**.033**^a^
Yes	*n* = 8 (20%)	*n* = 11 (29.73%)	*n* = 18 (47.37%)	
No	*n* = 32 (80%)	*n* = 26 (70.27%)	*n* = 20 (52.63%)	
Dosage (mg)	22.5 (39)	36.8 (86)	11.75 (14)	.263^b^
Frequency (day/year)	302.5 (208.5)	84 (128)	365 (317)	.091^b^
Iodine supplement intake				**.034**^a^
Yes	*n* = 5 (12.5%)	*n* = 4 (10.81%)	*n* = 12 (31.58%)	
No	*n* = 35 (87.5%)	*n* = 33 (89.19%)	*n* = 26 (68.42%)	
Dosage (μg)	75 (50)	70 (92.5)	150 (29.5)	.074^b^
Frequency (day/year)	180 (84)	242.5 (291)	365 (0)	**.016**^b^
Calcium supplement intake				**.002**^a^
Yes	*n* = 3 (7.5%)	*n* = 2 (5.41%)	*n* = 12 (31.58%)	
No	*n* = 37 (92.5%)	*n* = 35 (94.59%)	*n* = 26 (68.42%)	
Dosage (mg)	200 (210)	100 (0)	200 (300)	.385^b^
Frequency (day/year)	240 (182)	196.5 (337)	365 (91)	.651^b^
Eicosapentaenoic acid supplement intake				**.016**^a^
Yes	*n* = 5 (12.5%)	*n* = 3 (8.11%)	*n* = 12 (31.58%)	
No	*n* = 35 (87.5%)	*n* = 34 (91.89%)	*n* = 26 (68.42%)	
Dosage (mg)	220 (144)	225 (320)	217.5 (366.5)	.815^b^
Frequency (day/year)	156 (53)	365 (209)	365 (52.5)	.082^b^
Docosahexaenoic acid acid supplement intake				.052^a^
Yes	*n* = 6 (15%)	*n* = 4 (10.81%)	*n* = 12 (31.58%)	
No	*n* = 34 (85%)	*n* = 33 (89.19%)	*n* = 26 (68.42%)	
Dosage (mg)	185 (330)	441 (469)	354 (560)	.227^b^
Frequency (day/year)	143 (79)	260.5 (227)	365 (52.5)	.051^b^
Zinc supplement intake				.106^a^
Yes	*n* = 16 (40%)	*n* = 7 (18.92%)	*n* = 14 (36.84%)	
No	*n* = 24 (60%)	*n* = 30 (81.08%)	*n* = 24 (63.16%)	
Dosage (mg)	10 (10)	10 (5)	10 (3.14)	.498^b^
Frequency (day/year)	168 (329)	156 (335)	365 (182)	.065^b^
Magnesium supplement intake				.210^a^
Yes	*n* = 16 (40%)	*n* = 8 (21.62%)	*n* = 11 (28.95%)	
No	*n* = 24 (60%)	*n* = 29 (78.38%)	*n* = 27 (71.02%)	
Dosage (mg)	300 (220)	309.5 (296.7)	142 (178.5)	.252^b^
Frequency (day/year)	168 (309)	142 (259.5)	365 (268)	.534^b^
Selenium supplement intake				**.003**^a^
Yes	*n* = 3 (7.5%)	*n* = 4 (10.81%)	*n* = 13 (34.21%)	
No	*n* = 37 (92.5%)	*n* = 33 (89.19%)	*n* = 25 (65.79%)	
Dosage (μg)	25 (10)	55 (82.5)	55 (0)	**.021**^b^
Frequency (day/year)	240 (185)	92 (240.5)	365 (0)	.105^b^
Vitamin C supplement intake				.077^a^
Yes	*n* = 14 (35%)	*n* = 5 (13.51%)	*n* = 8 (21.05%)	
No	*n* = 26 (65%)	*n* = 32 (86.49%)	*n* = 30 (78.95%)	
Dosage (mg)	140 (260)	250 (125)	80 (100.23)	.493^b^
Frequency (day/year)	302.5 (209)	365 (0)	286.5 (261)	.545^b^
Folic acid supplement intake				.055^a^
Yes	*n* = 8 (20%)	*n* = 8 (21.62%)	*n* = 16 (42.11%)	
No	*n* = 32 (80%)	*n* = 29 (78.38%)	*n* = 22 (57.89%)	
Dosage (μg)	250 (200)	300 (343.75)	350 (200)	.847^b^
Frequency (day/year)	302.5 (210)	247.5 (291)	365 (169.5)	0.531^b^
Vitamin B1 supplement intake				.449^a^
Yes	*n* = 6 (15%)	*n* = 7 (18.92%)	*n* = 10 (26.32%)	
No	*n* = 34 (85%)	*n* = 30 (81.08%)	*n* = 28 (73.68%)	
Dosage (mg)	1.1 (2.3)	11.25 (25)	1.1 (2.2)	.283^b^
Frequency (day/year)	302.5 (182)	365 (337)	365 (0)	.148^b^
Vitamin B2 supplement intake				.110^a^
Yes	*n* = 5 (12.5%)	*n* = 7 (18.92%)	*n* = 12 (31.58%)	
No	*n* = 35 (87.5%)	*n* = 30 (81.08%)	*n* = 26 (68.42%)	
Dosage (mg)	1.4 (1.1)	11.4 (13.6)	1.4 (0.91)	.263^b^
Frequency (day/year)	365 (125)	365 (337)	365 (0)	.278^b^
Niacin supplement intake				.677^a^
Yes	*n* = 7 (17.5%)	*n* = 6 (16.22%)	*n* = 9 (23.68%)	
No	*n* = 33 (82.5%)	*n* = 31 (83.78%)	*n* = 29 (76.32%)	
Dosage (mg)	16 (32)	35.5 (29)	16 (0)	.406^b^
Frequency (day/year)	365 (185)	365 (235)	16 (0)	.328^b^
Vitamin B5 supplement intake				.417^a^
Yes	*n* = 5 (12.5%)	*n* = 6 (16.22%)	*n* = 9 (23.68%)	
No	*n* = 35 (87.5%)	*n* = 31 (83.78%)	*n* = 29 (76.32%)	
Dosage (mg)	6 (2)	18.125 (17)	6 (0)	.105^b^
Frequency (day/year)	365 (125)	274 (235)	365 (0)	.177^b^
Vitamin B6 supplement intake				.449^a^
Yes	*n* = 6 (15%)	*n* = 7 (18.92%)	*n* = 10 (26.32%)	
No	*n* = 34 (85%)	*n* = 30 (81.08%)	*n* = 28 (73.68%)	
Dosage (mg)	1.4 (3)	10.32 (18.6)	1.4 (2.2)	.256^b^
Frequency (day/year)	302.5 (185)	365 (337)	365 (0)	.151^b^
Biotin supplement intake				.604^a^
Yes	*n* = 6 (15%)	*n* = 8 (21.62%)	*n* = 9 (23.68%)	
No	*n* = 34 (85%)	*n* = 29 (78.38%)	*n* = 29 (76.32%)	
Dosage (μg)	50 (40)	31 (72.5)	50 (100)	.126^b^
Frequency (day/year)	302.5 (185)	156 (195)	365 (0)	**.016**^b^
Vitamin K1 supplement intake				.186^a^
Yes	*n* = 3 (7.5%)	*n* = 1 (2.70%)	*n* = 0 (0%)	
No	*n* = 37 (92.5%)	*n* = 36 (97.30%)	*n* = 38 (100%)	
Dosage (mg)	37.5 (1220)	30 (0)	–	.346^b^
Frequency (day/year)	52 (179)	365 (0)	–	.178^b^
Vitamin K2 supplement intake				.251^a^
Yes	*n* = 8 (20%)	*n* = 6 (16.22%)	*n* = 12 (31.58%)	
No	*n* = 32 (80%)	*n* = 31 (83.78%)	*n* = 26 (68.42%)	
Dosage (μg)	200 (85)	154.49 (140)	56.3 (37.5)	**.005**^b^
Frequency (day/year)	104 (266.5)	260.5 (209)	365 (52.5)	**.031**^b^
Vitamin E supplement intake				.755^a^
Yes	*n* = 4 (10%)	*n* = 2 (5.41%)	*n* = 3 (7.89%)	
No	*n* = 36 (96%)	*n* = 35 (94.59%)	*n* = 35 (92.11%)	
Dosage (mg)	9.25 (14.75)	66.6 (73.2)	12 (30.5)	.256^b^
Frequency (day/year)	210 (173.5)	365 (0)	365 (157)	.231^b^
Vitamin A supplement intake				**.026**^a^
Yes	*n* = 3 (7.5%)	*n* = 1 (2.70%)	*n* = 8 (21.05%)	
No	*n* = 37 (92.5%)	*n* = 36 (97.30%)	*n* = 30 (78.95%)	
Dosage (mg)	0.668 (0.6)	0.668 (0)	0.45 (0.225)	.666^b^
Frequency (day/year)	180 (287)	365 (0)	365 (0)	0.231^b^

^a^Based on Stata’s Chi-Square Test of independence.

^b^Based on Kruskal–Wallis *H* test.

**Table 4.** Laboratory measurements by dietary group.

	Omnivores (*n* = 40)	Lacto-Ovo-Vegetarians (*n* = 37)	Vegans (*n* = 38)	
White blood cell count (1000 cells/μL)	5.91 (2.07)^c^	5.16 (2.03)^d^	4.97 (1.63)^e^	0.258^a^
Platelet count (1000 cells/μL)	243 (74)^c^	230 (47)^d^	211 (73)^e^	0.070^a^
Red blood cell count (million cells/μL)	4.64 ± 0.36^c^	4.55 ± 0.48^d^	4.50 ± 0.46^e^	0.414^b^
Hemoglobin (g/dl)	13.71 ± 1.13^c^	13.46 ± 1.48^d^	13.51 ± 1.49^e^	0.726^b^
Hematocrit (%)	40.79 ± 3.11^c^	40.02 ± 3.66^d^	40.22 ± 3.76^e^	0.639^b^
Mean corpuscular volume (fL)	88.00 ± 3.17^c^	88.19 ± 3.60^d^	88.93 ± 4.48^e^	0.551^b^
Mean corpuscular hemoglobin (pg)	29.65 (2)^c^	29.6 (1.7)^d^	29.9 (2)^e^	0.066^a^
Mean corpuscular hemoglobin concentration (g/dl)	33.65 ± 0.86^c^	33.59 ± 1.14^d^	33.56 ± 1.17^e^	0.939^b^
Red cell distribution width	12.45 (0.7)^c^	12.6 (0.95)^d^	12.6 (0.7)^e^	0.499^a^
Neutrophil count (1000 cells/μL)	3.2 (1.78)^c^	2.75 (1.56)^d^	2.74 (1.38)^e^	0.637^a^
Lymphocyte count (1000 cells/μL)	1.83 (0.49)^c^	1.74 (0.57)^d^	1.53 (0.52)^e^	**0.018**^a^
Monocyte count (1000 cells/μL)	0.50 (0.13)^c^	0.48 (0.17)^d^	0.44 (0.11)^e^	0.052^a^
Eosinophils count (1000 cells/μL)	0.12 (0.11)^c^	0.12 (0.11)^d^	0.11 (0.08)^e^	0.788^a^
Basophils count (1000 cells/μL)	0.05 (0.02)^c^	0.04 (0.02)^d^	0.04 (0.02)^e^	0.257^a^
HbA1c (%)	5.25 ± 0.29^f^	5.20 ± 0.28^e^	5.07 ± 0.32^e^	**0.017**^b^
Vitamin B12 (pg/ml)	377 (225)^g^	310 (190)^e^	404.5 (195)f	**0.023**^a^
Holotranscobalamin 2 (pmol/l)	71.5 (63.6)^g^,^h^	40.1 (52.6)^e^^,^^h^	74 (87.2)f,h	**0.001**^a^
Folic acid (ng/ml)	9.1 (7.3)^g^,^i^	8.6 (5.9)^e^	10.85 (6.5)^f^	0.138^a^
Vitamin A (μg/l)	620 (250)^g^	560 (200)^e^	570 (190)^d^	0.546^a^
Vitamin B1 (μg/l)	56 (12)^g,j^	57 (14)^e^	57.5 (11)^f^	0.528^a^
Vitamin B2 (μg/l)	181 (35)^g^	184 (46)^e^	173.5 (29)^f^	0.637^a^
Vitamin B6 (μg/l)	34 (17)^g^,^j^	31 (14)^e^	35 (21)^f^	0.631^a^
Vitamin D (ng/ml)	27.00 ± 8.49^e^	29.46 ± 12.00^e^	33.38 ± 19.28^f^	0.144^b^
Vitamin E (μg/l)	16.44 ± 5.29^g^	13.75 ± 4.69^e^	13.89 ± 3.85^f^	**0.020**^b^
Vitamin K1 (μg/l)	0.43 (0.58)^g^	0.59 (0.61)^e^	0.64 (0.78)^f^	0.077^a^
Vitamin K2 (Menachinon-4, μg/l)	0.29 (0.11)^g^	0.28 (0.13)^e^	0.275 (0.08)^f^	0.792^a^
Vitamin K2 (Menachinon-7, μg/l)	0.26 (0.23)^g^,^k^	0.2 (0.15)^e^	0.36 (0.27)^f^	**0.012**^a^
Niacin (μg/l)	31 (8.6)^e^	29.2 (11.2)^e^	31.85 (9.1)^f^	0.705^a^
Albumin (g/dl)	4.74 ± 0.24^e^	4.78 ± 0.23^e^	4.80 ± 0.28^f^	0.646^b^
Blood urea nitrogen (mg/dl)	25 (6)^e^	22 (8)^e^	21 (9)^f^	**0.015**^a^
Cystatine C (mg/l)	0.84 (0.08)^e^	0.84 (0.11)^e^	0.87 (0.16)^f^	0.664^a^
Creatinine (mg/dl)	0.78 (0.17)^e^	0.73 (0.15)^e^	0.75 (0.17)^f^	0.036^a^
Iron (μg/dl)	63.95 ± 27.71^e^	62.76 ± 25.71^e^	71.66 ± 28.22^f^	0.309^b^
Ferritin (ng/ml)	42 (47)^e^	31 (52)^e^	35.5 (44)^f^	0.563^a^
Transferrin (mg/dl)	246 (42)^e^	262 (50)^e^	259.5 (56)^f^	0.625^a^
Transferring saturation (%)	18.11 ± 8.40^e^	17.57 ± 7.91^e^	19.63 ± 7.94^f^	0.519^b^
TSH (Thyroid-stimulating hormone; μU/ml)	2.1 (1.12)^e^	2.16 (1.01)^e^	2.07 (1.43)^f^	0.952^a^
Triiodothyronine (free T3; pmol/l)	4.86 ± 0.67^e^	4.57 ± 0.53^e^	4.72 ± 0.59^f^	0.130^b^
Thyroxine (free T4; pmol/l)	16.07 ± 2.40^e^	15.39 ± 1.98^e^	15.88 ± 2.27^f^	0.406^b^
Parathyroid hormone (pg/ml)	37 (15)^e^	37 (14)^e^	38 (15)^f^	0.83^a^
Glutamic oxaloacetic transaminase (U/l)	24 (7)^e^	22 (5)^e^	21 (6)^f^	0.361^a^
Glutamic-pyruvate-transaminase (U/l)	21 (12)^e^	18 (7)^e^	17.5 (9)^f^	**0.011**^a^
High-density lipoprotein (mg/dl)	54 (22)^e^	58 (16)^e^	55 (19)^f^	0.315^a^
Low-density lipoprotein (mg/dl)	106 (40)^e^	89 (37)^e^	79 (32)^f^	**0.001**^a^
Triglycerides (mg/dl)	82 (31)^e^	70 (34)	76 (35)	0.308^a^
High sensitive C-reactive protein (mg/l)	0.6 (1.7)e,k	0.4 (0.6)e,k	0.5 (0.7)f,k	0.093^a^
Selenium (μg/l)	90 (17)^l^	78 (18)^l^	75 (30)^m^	**0.007**^a^
Uric acid (mg/dl)	4.3 (1.3)^e^	4 (1)^e^	4.95 (1.3)^f^	**0.005**^a^

Normally distributed data is shown with its mean ± standard deviation; not normally distributed data is shown with its median and IQR in parenthesis.

^a^Based on Kruskal-Wallis H test.

^b^Based on analysis of variance.

^c^Based on *n* = 34 observations.

^d^Based on *n* = 36 observations.

^e^Based on *n* = 37 observations.

^f^Based on *n* = 38 observations.

^g^Based on *n* = 39 observations.

^h^Values >128 pmol/l which exceeded the reference range and which were no longer measurable were entered as 128 pmol/l into the analysis.

^i^Values >40 ng/ml which exceeded the reference range and which were no longer measurable were entered as 40 ng/ml into the analysis.

^j^Values >100 μg/ml which exceeded the reference range and which were no longer measurable were entered as 100 μg/ml into the analysis.

^k^Values <0,1 mg/l which exceeded the reference range and which were no longer measurable were entered as 0,1 mg/l into the analysis.

^l^Based on *n* = 34 observations.

^m^Based on *n* = 34 observations.

